# Rare Presentation of Left Lower Lobe Pulmonary Artery Dissection

**DOI:** 10.1155/2017/2760535

**Published:** 2017-01-05

**Authors:** René Hako, Ján Fedačko, Štefan Tóth, Radoslav Morochovič, Pavol Kristian, Tímea Pekárová, Petri Tuomainen, Daniel Pella

**Affiliations:** ^1^Department of Radiology, General Hospital Košice, 9 Masarykova, 040 01 Košice, Slovakia; ^2^First Department of Internal Medicine, Louis Pasteur University Hospital, Trieda SNP 1, 041 90 Košice, Slovakia; ^3^Department of Trauma Surgery, Faculty of Medicine, University of P. J. Šafarik, 43 Rastislavova, 040 01 Košice, Slovakia; ^4^Department of Infectology and Travel Medicine, Faculty of Medicine, University of P. J. Šafarik, 43 Rastislavova, 040 01 Košice, Slovakia; ^5^Department of Internal Medicine, Kuopio University Hospital and University of Eastern Finland, Kuopio, Finland

## Abstract

*Background*. Pulmonary arterial dissection with chronic pulmonary arterial hypertension as its major cause is a very rare but life-threatening condition. In most cases the main pulmonary trunk is the affected site usually without involvement of its branches. Segmental or lobar pulmonary artery dissection is extremely rare.* Case Presentation*. We report a unique case of left lower lobe pulmonary artery dissection in a 70-year-old male, with confirmed chronic pulmonary hypertension. To confirm dissection MDCT pulmonary angiography was used. Multiplanar reformation (MPR) images in sagittal, coronal, oblique sagittal, and curved projections were generated. This case report presents morphologic CT features of rare chronic left lobar pulmonary artery dissection associated with chronic pulmonary hypertension at a place of localised pulmonary artery calcification. CT pulmonary angiography excluded signs of thromboembolism and potential motion or flow artefacts.* Conclusion*. To the best of our knowledge, no case of lower lobe pulmonary artery dissection with flap calcification has been reported yet. CT imaging of the chest is a key diagnostic tool that is able to detect an intimal flap and a false lumen within the pulmonary arterial tree and is preferred in differential diagnosis of rare complications of sustained pulmonary arterial hypertension.

## 1. Background

Pulmonary arterial (PA) dissection represents a rare medical condition accompanied often with a sudden death. In most cases the final diagnosis is made only postmortem. The major complications of PA dissection leading to the death of the patient are cardiac tamponade and haemopericardium [1, 2]. Most common acute symptoms include dyspnoea, chest pain, and central cyanosis [3].

The major cause of PA dissection is a chronic PA hypertension. In most cases, it is associated with congenital cardiac abnormalities including patent ductus arteriosus [4] and double-outlet right ventricle [5]. Other rare causes include chronic inflammation of the pulmonary arteries, right heart endocarditis, amyloidosis, trauma, and severe atherosclerosis [6]. In some cases, the cause of pulmonary arterial dissection remains unknown [2, 7]. Pulmonary artery dissection with intimal flap calcification is very rare.

## 2. Case Presentation

A 70-year-old man with pulmonary hypertension presented to the emergency department with a two-day history of weakness accompanied with diarrhoea. He reported an episode of gripping upper chest pain 3 weeks ago, but an acute coronary syndrome was excluded at that time. Severe community acquired bacterial bronchopneumonia followed and it was treated with antibiotics. Past medical history showed he had undergone aortobifemoral bypass procedure in 2010. Physical examination revealed a regular pulse rate of 80 beats/min and a blood pressure of 100/70 mmHg. Cardiac auscultation revealed a loud pulmonary component of the second heart sound with parasternal heave. Respiratory sounds were normal. ECG displayed atrial flutter and biventricular hypertrophy. Cardiac enzymes and D-dimer levels were within normal range. Chest X-ray displayed moderate cardiomegaly with an enlarged pulmonary trunk and pulmonary vessels. No abnormalities were seen in lung parenchyma and pleura.

Echocardiogram demonstrated normal left ventricular systolic function with moderate hypertrophy, moderate mitral regurgitation, atrial septal defect (*ostium secundum*), dilated and severely hypertrophied right ventricle with impaired function, moderate biatrial enlargement, and a dilated main pulmonary artery (65 mm). Systolic pulmonary artery pressure was 64 mmHg, which suggests severe pulmonary hypertension.

After abdominal aorta ultrasound examination, aortobifemoral bypass thrombosis with mesenteric ischaemia was assumed to be the cause of diarrhoea. Contrast-enhanced abdominal CT angiography was performed but no thrombosis of aortobifemoral graft or superior/inferior mesenteric artery was identified. The CT scan of lower chest accidentally showed an intimal dissection flap in the lower lobe of the left pulmonary artery.

To confirm dissection, MDCT pulmonary angiography was performed using a Somatom Sensation Open 64 CT scanner (Siemens, Germany). We acquired MDCT data during an IV injection of 100 ml of the iodinated contrast agent iomeprol (Iomeron 400, Bracco Imaging, Germany) at a rate of 4 mL/sec. The following scanning protocol was used: collimation, 4 × 1 mm; gantry rotation time, 500 msec; and table feed, 1.5 mm/rotation. The tube current was 150 mA at 140 kV to keep the radiation dose within a reasonable range. The scan delay was determined using bolus tracking. Multiplanar reformation (MPR) images in sagittal, coronal, oblique sagittal, and curved projections were generated.

**Figure 1:**
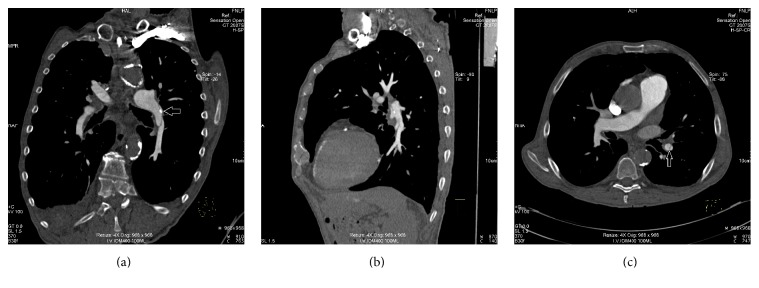
Coronal and sagittal curved MPR section (a, b) of a contrast-enhanced CT pulmonary angiogram showing long thin dissection flap in the lower lobe branch of the left pulmonary artery with partial involvement of the left pulmonary artery with a small intimal flap calcification (white arrow) and axial section with partial involvement of the lower segmental branch (white arrow) (c).

CT pulmonary angiography excluded signs of acute or chronic thromboembolism and potential motion or flow artifacts but confirmed a 55 mm long dissection flap in the lower lobe branch of the left pulmonary artery with partial involvement of the left pulmonary artery with a small intimal flap calcification (6 × 3 mm) and with partial involvement of the lower segmental pulmonary artery branch (Figure 1). After the diagnosis was made, the patient refused any interventional approach and died a week later.

## 3. Conclusions

Development of an intimal tear in pulmonary vessels can be spontaneous or caused by the pulmonary catheter. Pathophysiology of spontaneous pulmonary artery dissection during pulmonary hypertension is still not clear. There are several suggested conditions associated with the PA dissection development. Medial degeneration of large elastic pulmonary arteries with elastic fibre fragmentation and marked PA dilatations are the strongest predisposing factors. In pulmonary hypertension, dissection most often occurs at the site of a pulmonary aneurysm or a marked dilatation [8]. It is reasonable to assume that a spontaneous dissection occurs at the point where pulmonary artery tissue becomes too fragile to support the tension of the pulmonary artery wall. In addition to these conditions, preexisting local inflammation of the pulmonary vessels caused by thrombosis may also be related to the formation of intimal tear [9].

For the diagnosis of PA dissection, the following imaging methods are available: transthoracic echocardiogram (image of main PA dilatation with intimal flap [10]), CT, magnetic resonance imaging, and pulmonary angiography [11]. CT scans and magnetic resonance imaging of the chest are key diagnostic tools that are able to detect an intimal flap and a false lumen within the pulmonary arterial tree and are able to evaluate the extensiveness of the lesions [8]. Complications of sustained pulmonary arterial hypertension that are detectable with CT include central pulmonary artery embolism, premature atherosclerosis of the pulmonary arteries, pulmonary artery dissection, and right heart chamber hypertrophy and dilatation [11]. It was suggested that a pseudoflap in the pulmonary artery may represent motion artefact from aortic and cardiac pulsation motion and thus could lead to false diagnosis of pulmonary artery dissection [8]. Dissection in the main pulmonary trunk is an extremely rare but usually lethal complication of chronic pulmonary hypertension [12]. The main pulmonary trunk is the affected site in 80% of the cases, usually without involvement of its branches. Recent isolated reports have described pulmonary artery dissection in surviving patients [1].

Symptoms associated with pulmonary artery dissection include chest pain, fatigue, and progressive dyspnoea. Dissection of PA often manifests as cardiogenic shock or sudden death [13]. Sudden death occurs most commonly due to cardiac tamponade as the vessel dissects into the pericardium. In that series, chest pain occurred in 67%, dyspnoea in 82%, and central cyanosis in 52% of the cases [1].

Treatment of pulmonary artery dissection is surgical in case of symptomatic patients [14]. Nonoperative treatment is reserved for stable asymptomatic patients [15, 16].

Since the 1st description by Walshe in 1862, only about 50 cases of pulmonary artery dissection have been reported [17]. Only seven previous cases have had radiological evidence of pulmonary artery dissection [6]. To the best of our knowledge, only 3 previous cases have had radiological evidence of segmental or lobar pulmonary artery dissection, and only one case of left lobar pulmonary artery dissection has been reported [12]. This report documents case of left lower lobe pulmonary artery dissection with unusual small flap amorphous calcification. Intimal pulmonary artery calcifications are extremely rare. Increased attention has focused on the role of inflammation in the development of atherosclerosis and vascular calcifications. Thus, it is possible that both vascular calcifications and atherosclerosis are inflammation-dependent processes [18].

Lobar PA dissection symptoms often mimic aortic dissection or MI. In comparison with the dissection of pulmonary trunk, lobar PA dissection is less malignant in progression and has a significantly longer survival even without the treatment. Pulmonary artery dissection should be considered in patients with chronic pulmonary hypertension and manifestation of the chest pain. Echocardiography is still the first line of investigation due to its accessibility. It can be used for rapid exclusion of other causes of chest pain. CT imaging methods provide important additional anatomical information of the pulmonary arterial tree mainly in the cases where echocardiography shows no clear evidence but also cannot exclude the PA dissection.

## Additional Points

*Advances in Knowledge*. We report an unusual case of left lower lobe pulmonary artery dissection with flap calcification; to the best of our knowledge, there has been only one case previously reported in the scientific literature. No case of lower lobe pulmonary artery dissection with flap calcification has been reported.
